# Ovarian Seromucinous Tumors: Pathogenesis, Morphologic Spectrum, and Clinical Issues

**DOI:** 10.3390/diagnostics10020077

**Published:** 2020-01-31

**Authors:** Michiko Nagamine, Yoshiki Mikami

**Affiliations:** 1 Department of Pathology, Japanese Red Cross Kumamoto Hospital, Nagamineminami, Kumamoto 861-8520, Japan; 2 Department of Diagnostic Pathology, Kumamoto University Hospital, Chuo-ku, Kumamoto 860-8556, Japan; mika@kuhp.kyoto-u.ac.jp

**Keywords:** seromucinous tumor, endocervical-like mucinous borderline tumor, ovarian epithelial tumor

## Abstract

Ovarian seromucinous tumors were introduced in the 2014 World Health Organization (WHO) classification as one of the seven types of ovarian epithelial tumors. They are characterized by frequent association with endometriosis and bilaterality, microscopic appearance of papillary architecture, and admixture of a variety of müllerian-type epithelium. They are considered to be endometriosis-related ovarian neoplasms, along with endometrioid and clear cell tumors; recent molecular studies suggest this particular tumor is a variant of endometrioid tumor. Discrepancies in nomenclature, definition, and morphology of seromucinous tumors appear to be a source of confusion, for both clinicians and general surgicalpathologists. This review summarizes the clinicopathological features of benign, borderline, and malignant seromucinous tumors, as well as controversies regarding these tumors.

## 1. Introduction

In 1988, Rutgers and Scully first described two “closely related” ovarian borderline tumors, i.e., müllerian mucinous papillary borderline tumor (MMBT) [1] and mixed-epithelial papillary borderline tumor of müllerian type (MEBT-M) [2]. These two tumors shared the morphology of papillary architectures similar to those of serous borderline tumor (SBT). However, MEBT-M was distinguished from MMBT by the presence of two or more müllerian type cells, each accounting for at least 10%, whereas MMBT was predominantly composed of endocervical-type, mucin-producing columnar cells. They defined müllerian cell types to include serous-, mucinous-, endometrioid-, and squamous-type cells. In addition, “indifferent cells” with abundant eosinophilic cytoplasm were also described as components of both MMBT and MEBT-M. Subsequently, the 2003 World Health Organization (WHO) classification included MMBT as an endocervical-like mucinous borderline tumor (EMBT) and also MEBT-M in the mixed-epithelial tumor category [3].

The term “seromucinous” has been used among gynecologic pathologists for over 40 years to describe a subset of ovarian epithelial tumors [4], but it drew much attention when the 2014 WHO classification adopted seromucinous ovarian tumors as a new category, distinct from mucinous tumors which show gastrointestinal differentiation [5]. Ovarian epithelial tumors are now divided into seven types: serous, mucinous, endometrioid, clear cell, Brenner, undifferentiated (carcinoma), and seromucinous. The mixed epithelial tumor category has been eliminated. Alternatively, seromucinous tumors were defined as epithelial tumors composed of two or more müllerian cell types; seromucinous borderline tumor (SMBT) is thus a synonym for MEBT-M, MMBT, and EMBT.

This review describes the pathological and clinical characteristics of seromucinous tumors. We also discuss current concepts and controversies regarding these tumors.

## 2. Morphologic Spectrum

Seromucinous tumors are commonly borderline, but benign and malignant categories do exist [1,6]. The common characteristic features of all categories of seromucinous tumors are (i) frequent involvement of both ovaries (20−40%), (ii) association with endometriosis (30−70%), and (iii) absence of gastrointestinal differentiation, as evidenced by admixtures of goblet cells and/or Paneth cells [7,8]. Evidence of gastrointestinal differentiation makes the diagnosis of seromucinous tumor unlikely.

### 2.1. Seromucinous Cystadenoma/Adenofibroma

**Figure 1:**
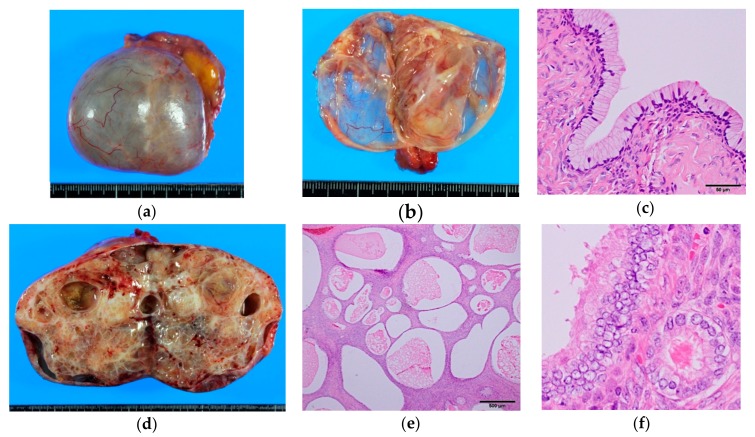
Gross and microscopic appearance of benign seromucinous tumors. (**a**–**c**) Seromucinous cystadenoma. A thin-walled paucilocular cyst filled with thin mucinous clear fluid. The cyst wall is lined by endocervical-type columnar mucinous cells admixed with scattered ciliated cells. Note the resemblance to normal endocervical epithelium (Figure 6a); (**d**–**f**) Seromucinous adenofibroma. Mostly solid mass with scattered cystic spaces. Endocervical-type cells proliferate in an adenofibromatous pattern. Focal nuclear enlargement and stratifications—but no papillary projections—are seen.

These benign tumors account for about 1% of ovarian benign epithelial neoplasms [5]. They are usually cystic but can be adenofibromatous (Figure 1). Cystic lesions are unilocular or oligolocular with smooth outer and inner surfaces. The cyst fluid can be serous or mucinous. Typically, endocervical-type columnar cells with abundant intracytoplasmic mucin line the inner surface, but there also is a variable admixture of ciliated cells, endometrioid cells, and less often, transitional or squamous cells. By definition, as mentioned above, they should consist of two or more of these cell types, each comprising greater than 10% of the lesion, although prototypically, the tumor is composed of purely endocervical-type epithelium. Applying the same criteria with serous and mucinous tumors, focal epithelial proliferation or nuclear enlargement, as seen in SMBTs, comprising less than 10% of the whole tumor, is best designated as benign (“seromucinous cystadenoma with focal atypia”) [7]. Distinguishing an endometriotic cyst with mucinous or ciliated metaplasia is somewhat subjective, but existence of endometrial stroma and focal nature of the mucinous/ciliated changes support a diagnosis of endometriotic cyst.

### 2.2. Seromucinous Borderline Tumor (SMBT)

**Figure 2:**
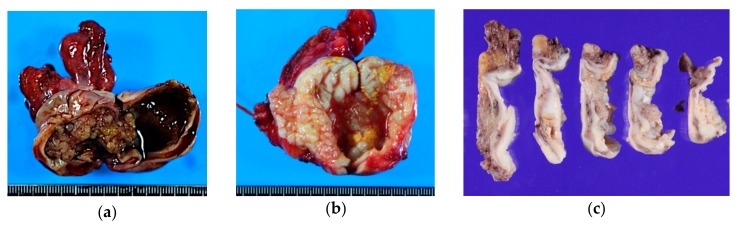
Gross appearance of seromucinous borderline tumor. They usually present as relatively small, unilocular cysts. (**a**) Seromucinous borderline tumor (SMBT) is often seen in a background of an endometriotic cyst; (**b**,**c**) Unilocular cyst containing serosanguinous fluid with papillary projections on the inner surface. Note the thick, fibrous cyst wall on the multi-sectioned cut surfaces (**c**).

The average age of patients is 34–44 years. These tumors affect ovaries bilaterally in up to 40% of patients [9]; 30–50% are associated with endometriosis [7,10]. Grossly, they are unilocular or oligolocular cysts with papillary projections on the inner surface (Figure 2). The cyst wall is often fibrous and thickened. The mean size is 8–10 cm. Cyst content can be hemorrhagic, serous, or mucinous, and frequently mucopurulent, due to infiltration of neutrophils. Mural nodules, as seen in gastrointestinal-type MBT, is exceedingly rare in SMBT.

Microscopically, SMBT is characterized by a papillary structure with hierarchical branching, with typically bulbous and edematous, sometimes sclerotic, stroma. Numerous neutrophils are commonly observed in stroma, epithelium, or luminal spaces. Eosinophilic infiltration can also be seen [11].

**Figure 3:**
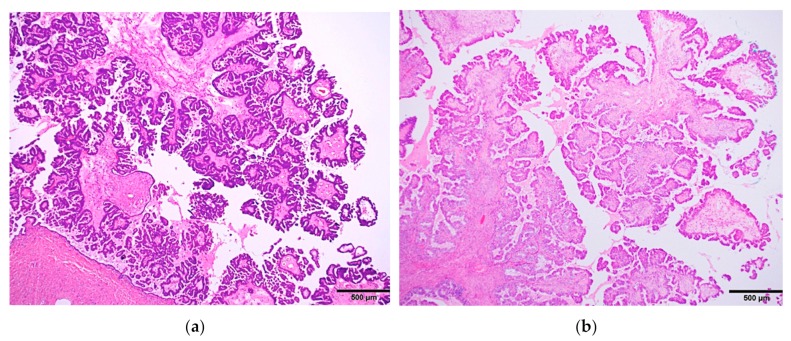
Low-power microscopic pictures showing hierarchical papillary structures of two entirely different tumors: (**a**) serous borderline tumor and (**b**) seromucinous borderline tumor. Note the striking resemblance between the two.

**Figure 4:**
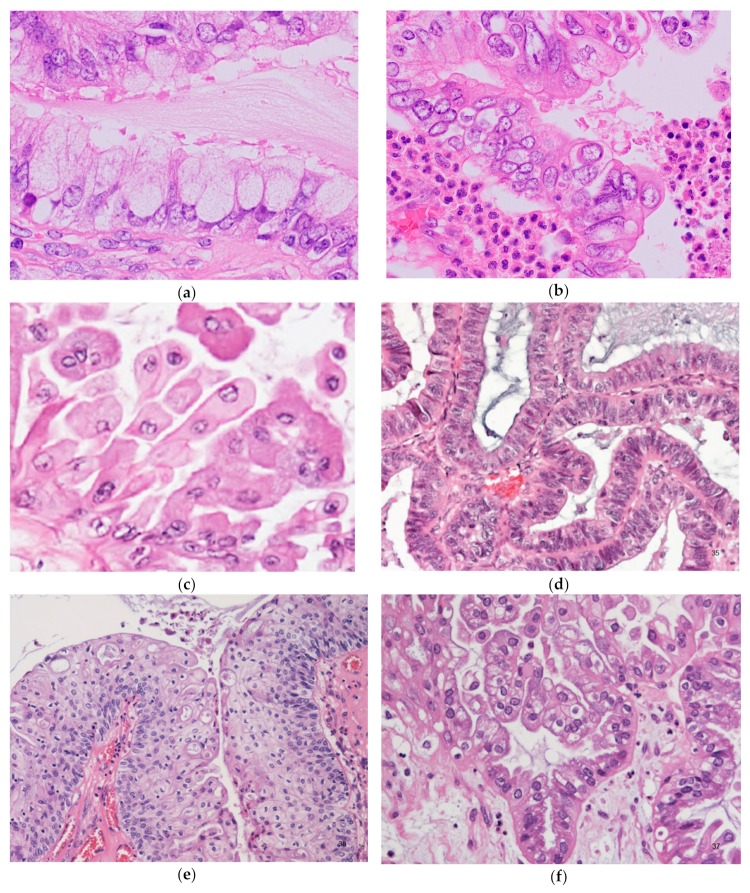
Seromucinous borderline tumor; microscopic pictures showing a variety of cell types. (**a**) Sometimes mucinous cells with voluminous cytoplasm can mimic goblet cells. Admixed lightly eosinophilic ciliated cells are almost always identifiable. (**b**) Background of prominent neutrophilic infiltration. Admixture of mucinous cells, eosinophilic cells, and some clear cells, with mild or moderate nuclear atypia and stratifications. (**c**) Indifferent cells with abundant eosinophilic cytoplasm. (**d**) Endometrioid type epithelium. (**e**) Squamous epithelium. (**f**) Clear cells. Focal hobnail appearance is also seen (right lower corner).

Although the low-power architecture closely resembles SBT (Figure 3), the lining cells are distinctly different from SBT, composed of a variety of epithelial cells (two or more cell types by definition), most commonly including endocervical-type mucinous, ciliated, endometrioid, and indifferent eosinophilic cells; and less commonly, squamous, clear, and hobnail cells (Figure 4).

Proportions of these cells are highly variable. Sometimes squamous epithelium predominates [12]. Nearly all tumors show, at least focally, indifferent eosinophilic cells (Figure 4c). These cells are stratified polygonal cells with abundant eosinophilic cytoplasm and centrally located nuclei, typically located at the tips of papillae. Microglandular hyperplasia-like areas may also be seen [2], and occasionally, subepithelial cuboidal cells, immunohistochemically positive for p63 and high molecular weight cytokeratin, which resemble reserve cells of the uterine cervix, may be identified [13]. High-grade nuclear atypia without destructive invasion is interpreted as intraepithelial carcinoma. Frank stromal invasion of < 5 mm in greatest dimension is defined as microinvasion, regardless of number of foci; tumors with microinvasion are considered SMBT (“SMBT with microinvasion”).

Immunohistochemically, epithelial cells are positive for CK7, PAX8, estrogen receptor (ER), progesterone receptor (PR), and negative for WT-1, CK20, CDX-2 [14]. WT-1 is negative even in the area of “serous” type epithelium [15]. In contrast, SBT is almost always positive for WT-1; and MBT is variably positive for CK20 and CDX-2, while negative for ER and PR. From the practical point of view, the diagnosis is almost always straightforward, but distinction between SBT and SMBT can be a matter; thus, WT-1 may be used in daily practice.

Up to 90% of cases present as FIGO stage I, and a subset of cases show extraovarian disease. Only three cases of stage IV disease have been reported in the literature [12,16,17], but the prognosis is excellent even for advanced-stage disease [18]. In only one out of the 380 cases of SMBT reported in the literature did the patient die of the disease, which had recurred as carcinoma [7,17,19].

Extraovarian disease does not show features of pseudomyxoma peritonei, as seen in cases of MBT, and are typically present as peritoneal implant. The implants may elicit desmoplastic stromal reactions on the peritoneal surface, and embedded clusters of mucin-producing tumor cells may be misinterpreted as disseminated adenocarcinoma on frozen section during intraoperative consultation. Lymph node involvement can also be present. Both conditions, diagnosed using the same criteria used for SBT, are identified in 10–20% of cases.

Patients who are initially treated with cystectomy, particularly those of reproductive age, can be managed by conservative observation if no residual disease is identified by the imaging studies. However, patients should be strongly informed of their risk of recurrence or involvement of the contralateral ovary and require a long-term follow-up.

### 2.3. Seromucinous Carcinoma

**Figure 5:**
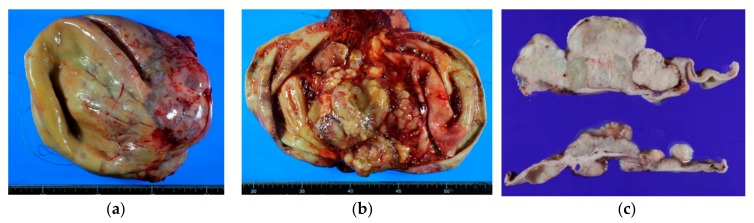
Gross features of seromucinous carcinoma. (**a**–**c**) Relatively large, unilocular cyst containing thin mucinous hemorrhagic fluid. Solid growth on inner surface is well appreciated on the cut surfaces.

Seromucinous carcinoma (SMCA) is relatively rare; fewer than 40 cases have been described in the English literature, and no stage IV disease has been reported yet [20,21,22]. SMCA shares gross and microscopic features with SMBT (Figure 5), demonstrating papillary projections resembling those of SBT and admixture of several müllerian cell types, and shows high-grade cytologic atypia and architectural complexity. Cribriform and solid growth patterns are commonly seen, and patterns of stromal invasion are divided into expansile and are destructive as in mucinous carcinoma. The mitotic index varies but tends to be low, usually less than 5/10 HPF [5].

Due to limited experience, the small number of reported cases, and possible inter-observer variability on diagnosis (as mentioned below), the exact incidence and prognosis of SMCA are unclear. One study found SMCA comprised 4% (6/149 cases) of all ovarian carcinomas, with lower 5-year survival (55%) than for clear cell carcinoma (75%); however, the difference was not statistically significant [22].

## 3. SMBT as an Endometriosis-Related Neoplasm (ERON)—Molecular Abnormalities

In 2010, two groups almost simultaneously reported somatic *ARID1A* (AT-rich interactive domain 1A gene) mutations in 57% and 46% of clear cell carcinomas, respectively [23,24]. This abnormality first appeared to be specific for clear cell carcinoma but was also found in 30% of ovarian endometrioid carcinomas, and none of the high-grade serous carcinomas [23]. These facts indicate that *ARID1A* mutation is a major genetic alteration in ERONs [25,26,27]. *ARID1A* is a tumor suppressor gene located at 1p36.11 and encodes the BAF250a protein. BAF250a is a member of the SWI/SNF (SWItch/Sucrose Non-Fermentable) family, which plays an important role in chromatin remodeling. Importantly, *ARID1A* mutation and protein loss are also identified in atypical endometriosis adjacent to clear cell carcinoma, which suggests that this abnormality is an early event in endometriosis-related carcinogenesis [23,27].

Subsequently, *ARID1A* protein loss was identified by immunohistochemistry in eight of 24 cases, and somatic mutation in two of two cases, of SMBT [10]. The association between endometriosis and *ARID1A* mutation indicates that seromucinous tumors are a type of ERON, along with endometrioid and clear cell tumors [18,25]. Based on the observation of coexistence of SMCA and SMBT, some gynecologic pathologists believe SMCA is a type I ovarian carcinoma that arises from its benign counterpart through a borderline tumor [28]. These concepts are of importance in developing strategy for early detection of SMCA among patients with endometriosis.

*KRAS* mutation is identified in 69% of SMBT in exon 1 and codon 12 [29]. On the other hand, *PTEN* mutation is not common in SMBT; both mutational analysis [29] and immunohistochemical study [15] failed to demonstrate *PTEN* abnormalities in this tumor. Data on genetic alterations in seromucinous carcinoma are limited, although one international study showed mutations in *ARID1A, KRAS*, *PIK3CA*, and *PTEN* in 16%, 70%, 37%, and 19% of seromucinous carcinoma, respectively, based on analysis of 32 cases [21]. These results call the existence of seromucinous carcinoma as a distinct entity into question, as discussed below.

## 4. Confusion and Controversies

### 4.1. Nomenclature Confusion

**Figure 6:**
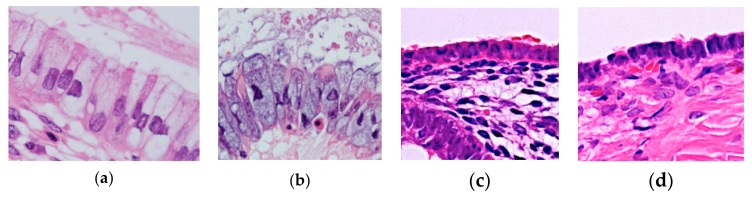
(**a**) Normal endocervical epithelium. (**b**) SMBT. (**c**) Normal surface endometrial epithelium of the uterus. (**d**) Serous cystadenoma. Scattered ciliated cells are admixed in all of the four epithelia. Notice the similarity between (**a**,**b**) and between (**c**,**d**), which are indistinguishable on hematoxylin-eosin stain.

The term “seromucinous tumor” has been a source of confusion because it sounds as if this tumor is composed of a mixture of serous tumor and mucinous tumor, which is not true. A prototypical seromucinous tumor, mostly borderline, is an epithelial tumor that shows endocervical glandular differentiation. Importantly, normal endocervical glandular epithelium is composed of mucin-secreting columnar epithelium with pale and rather basophilic cytoplasm (in contrast to intestinal- or gastric-type mucous cells), and it contains ciliated cells with slender and eosinophilic cytoplasm. The epithelia of seromucinous tumors mostly resembles the endocervical glandular epithelium; in this regard, the term “endocervical-like” is appropriate. In fact, in the 2003 WHO classification, EMBT (currently, a synonym for SMBT) was defined as a tumor of low malignant potential, composed of mucinous epithelial cells that resemble endocervical epithelium [3]. Kurman et al. [4] also claimed that “seromucinous” tumors are neither serous tumors nor mucinous tumors, based on clinical, immunohistochemical, and molecular findings; they also pointed out that merely having ciliated cells does not qualify as serous differentiation, as endometrium can show similar changes (Figure 6).

As SMBT/EMBT comprises a variety of other müllerian-type cells, the term “Müllerian tumors of mixed cell type, borderline” was in fact considered for the 2014 WHO classification as an alternative for SMBT/EMBT, which was defined as “a noninvasive proliferative epithelial tumor of more than one epithelial type most often serous and endocervical-type mucinous, sometimes endometrioid, and less often clear cell, transitional or squamous.” However, in the absence of sufficient discussion and consensus, the term “seromucinous” was finally adopted, with its definition retained; thus, the term SMBT is currently used worldwide. The definition and absence of quantitative criteria, i.e., the “10% rule,” are primary sources of confusion, resulting in discrepancy between its name, definition, and morphology. For example, a borderline ovarian epithelial tumor composed of 20% endocervical type mucinous, 20% serous, and 60% endometrioid components, is regarded as SMBT. Therefore, “mixed müllerian tumors” or “müllerian mucinous/mixed epithelial tumor” is preferred among some gynecologic pathologists [7,8].

### 4.2. Seromucinous Carcinoma as a Distinct Entity

Seromucinous carcinoma and endometrioid carcinoma with mucinous differentiation, commonly endocervical-like, show overlapping morphology, and their distinction is arbitrary. In fact, some gynecologic pathologists regard seromucinous carcinoma as a variant of endometrioid carcinoma. In addition, results from recent molecular studies cast doubt on the existence of seromucinous carcinoma. Rambau et al. defined histotype-specific immunophenotypes and genotypes for representative ovarian carcinomas, and conducted a multi-institutional study [21]. They examined 32 cases of seromucinous carcinoma and demonstrated suboptimal interobserver reproducibility for diagnoses; all tumors were re-categorized as either endometrioid (23 cases), low-grade serous (8 cases), or mucinous (1 case) carcinomas. Their results imply that the term “seromucinous carcinoma” should be abandoned.

In these authors’ opinion, mere genotype does not necessarily define an entity, and histogenesis, morphology, and consistency of terminology should also be considered in tumor typing. In addition, prototypical SMBT occasionally contains intraepithelial carcinoma and may coexist with carcinoma. Such carcinoma is semantically considered to be “seromucinous carcinoma,” and may be distinguished from endometrioid carcinoma that shows mucinous differentiation without a borderline component. However, absence of consensus between gynecologic pathologists with regard to diagnostic criteria for seromucinous carcinoma, as well as consequent confusion among gynecologists, justifies the removal of this category from classification. This particular tumor may just be regarded as a variant of endometrioid carcinoma.

## 5. Conclusions

Over the past three decades, numerous clinicopathologic and molecular studies have been published to delineate seromucinous tumors. The wide morphological spectrum of SMBT has been a source of confusion, although the prototypical case is characterized by endocervical-like morphology and phenotype. Seromucinous carcinoma, as a distinct entity, has been also questioned; its distinction from endometrioid carcinoma with mucinous differentiation is particularly controversial. Clearly, we need more information to solve these issues, and we hope the future journey will be as exciting as the one we have experienced in the past 30 years.
